# Seroprevalence and risk factors of bluetongue virus infection in sheep and goats in West Gondar zone, Northwest Ethiopia

**DOI:** 10.3389/fvets.2025.1565624

**Published:** 2025-03-05

**Authors:** Adem Beyan, Wassie Molla, Adugna Berju Molla, Mastewal Birhan, Saddam Mohammed Ibrahim, Bereket Dessalegn, Ambaye Kenubih, Abebe Tesfaye Gessese, Mebrie Zemene Kinde, Gashaw Getaneh Dagnaw, Melkie Dagnaw Fenta, Hana Tesfaye, Takele Tesgera, Liyuwork Tesfaw, Habtamu Abesha, Zewdu Seyoum Tarekegn, Haileyesus Dejene, Molalegne Bitew

**Affiliations:** ^1^Department of Veterinary Epidemiology and Public Health, College of Veterinary Medicine and Animal Sciences, University of Gondar, Gondar, Ethiopia; ^2^Department of Veterinary Pathobiology, College of Veterinary Medicine and Animal Sciences, University of Gondar, Gondar, Ethiopia; ^3^Department of Veterinary Biomedical Sciences, College of Veterinary Medicine and Animal Sciences, University of Gondar, Gondar, Ethiopia; ^4^Department of Veterinary Clinical Medicine, College of Veterinary Medicine and Animal Sciences, University of Gondar, Gondar, Ethiopia; ^5^Department of Veterinary Pharmacy, College of Veterinary Medicine and Animal Sciences, University of Gondar, Gondar, Ethiopia; ^6^Department of Animal Health and Quality Control Team, National Veterinary Institute, Bishoftu, Ethiopia; ^7^Metekel Zone Agriculture and Rural Development Office, Metekel, Ethiopia; ^8^Health Biotechnology Directorate, Bio and Emerging Technology Institute, Addis Ababa, Ethiopia

**Keywords:** bluetongue virus, goat, risk factors, seroprevalence, sheep, West Gondar

## Abstract

**Introduction:**

Bluetongue is a non-contagious arthropod-borne viral disease that affects ruminants. No investigations have yet been conducted to ascertain the seroprevalence and associated risk factors of bluetongue in Northwest Ethiopia. It is essential to determine the seroprevalence and correlated risk elements to formulate an effective strategy for preventing and surveillance of the disease.

**Methods:**

A cross-sectional study was carried out between February 2023 and May 2023 to determine the seroprevalence and risk factors associated with Bluetongue virus (BTV) in sheep and goats in the selected districts of West Gondar zone. A multistage cluster sampling technique was employed, with zones and districts purposively selected, and kebeles within these districts chosen through simple random sampling. Villages were treated as clusters. A total of 444 blood specimens were collected from the sheep and goats and subsequently tested for BTV antibodies using a commercially available competitive enzyme-linked immunosorbent assay kit. A mixed-effects logistic regression was employed to evaluate the relationship between Bluetongue virus seropositivity and potential risk factors.

**Results:**

The overall seroprevalence rate at the individual animal level was 84.5% (95% CI: 81.09–87.82). The seroprevalence in sheep and goats was 83.8% (257/308) and 86.8% (118/136), respectively. Species and age were significant risk factors for BTV seropositivity in the study area (*p* < 0.05). Adult and older sheep and goats exhibited 3.49 (95% CI: 1.90–6.41) and 25.95 (95% CI: 9.45–71.28) times higher seroprevalence with the bluetongue virus in comparison to their younger counterparts, respectively.

**Discussion:**

In conclusion, the current findings showed that BTV is highly prevalent. The specific circulating BTV serotypes and the temporal pattern of Bluetongue in the study area remain unknown, necessitating further investigation.

## 1 Introduction

Bluetongue (BT) is a non-contagious viral disease transmitted by arthropods that affects both domestic and wild ruminants, particularly in tropical and subtropical regions. It represents a significant vector-borne viral infection (1–3) caused by the Bluetongue virus (BTV) of the Orbivirus genus within the Reoviridae family. Currently, 29 known serotypes of BTV exist, with ongoing discoveries of novel types (4–7). Recent serotypes such as BTV-25, BTV-26, and BTV-27 are believed to be transmitted exclusively through vector-independent pathways, potentially leading to persistent infection in goats (8–10). The impact of BTV infection includes severe direct economic consequences (11), alongside indirect financial losses due to trade restrictions and reduced animal productivity. In Ethiopia, the disease poses substantial economic challenges, directly resulting in livestock mortality, decreased milk production, weight loss, and reproductive disorders. Indirectly, trade restrictions limit export opportunities, adversely impacting livestock-dependent farmers by reducing household income and constraining economic growth (12, 13). The molecular assays have revealed the existence of two major ancestral lineages: a Western lineage (found in Africa, Europe, and the Americas) and an Eastern lineage (found in Australia and Asia) (5, 14–16).

BTV is widely recognized as an endemic disease in Africa, yet there is a scarcity of data on its prevalence across most nations on the continent (17). BTV epidemics are often associated with periods of intense rainfall. Outbreaks affecting cattle, sheep, and goats have been reported in North and East Africa, particularly in Egypt, Algeria, Tunisia, and Kenya (18, 19). In Southern Africa, incidents involving sheep and goats have been documented in Botswana, Lesotho, Madagascar, Namibia, South Africa, and Zimbabwe (20–23). However, comprehensive data on the prevalent serotypes is available only for South Africa (serotypes 1–24) and Malawi (serotypes 1, 2, 3, 5, 8, 10, 15, 20, 21, and 22) (21). Additionally, since the late 20th century, multiple outbreaks of BT have been documented across European countries, with BTV strains predominantly believed to follow a consistent transmission route from North Africa to Southern Europe (2, 3, 24–26). Moreover, in Ethiopia, research on BTV epidemiology has predominantly focused on the southwestern regions, particularly around Jimma, Bonga, Bedele by Abera et al. (13), and the Maji districts by Haile et al. (12), with only a handful of studies conducted on the subject.

Midges belonging to an expanding array of species within the *Culicoides* genus (27) typically serve as vectors for BTV among susceptible ruminants (9, 28–30). In Northwest Ethiopia, a study by Ayele et al. (31) on *Culicoides* identification documented twelve species, eight of which, *C. corsicus, C. kibunensis, C. reioxi, C. kiouxi, C. saharienines, C. desertorum, C. reithi*, and *C. festivipennis*, had not been previously recorded in Ethiopia. Moreover, the seasonality of bluetongue infection is closely linked to the dependency of *Culicoides* on climatic variations. These midges breed in moist environments such as streams, irrigation channels, muddy areas, and regions with fecal runoff around farms, with suitable habitats being prevalent in many farming settings (9, 32–34). Cattle, acting as reservoirs and amplifying hosts, exhibit high levels of viremia.

The initial identification of BTV dates back to the late 18th century in South Africa, following the importation of prized fine wool sheep from Europe (16, 17, 20, 34). Presently, bluetongue disease is found on all continents except Antarctica, with various serotypes and strains leading to diverse disease manifestations (12). Recent studies by Gulima (35), Gizaw et al. (1), Yilma and Mekonnen (36), Abera et al. (13), and Haile et al. (12) reported seroprevalence rates of 34.1% in Amhara region, 65.2% in Oromia region, Afar region, South Nation and Nationality Regional State (SNNPRS), and Somalia region, 41.17% in SNNPRS, 30.6% in Oromia region, and 39.23% in SNNPRS, respectively. The Amhara region, which boasts a sheep population of 10,391,582 and a goat population of 7,045,305, ranks second and fourth in Ethiopia, respectively. Annually, in the Amhara region, 1,541,624 sheep and 1,239,994 goats succumb to diseases, marking the second and third highest mortality rates in the country after poultry (37); however, the specific contribution of bluetongue to these figures remains unclear. Despite the prevalence of the disease in the region, no investigations have been conducted to ascertain the seroprevalence and associated risk factors of bluetongue in sheep and goats in Amhara region, Northwest Ethiopia. Therefore, conducting research on these aspects is crucial for generating valuable data to guide future studies and to aid in the development of effective disease control and prevention strategies. The primary objective of this study was to determine the seroprevalence of bluetongue virus infection in sheep and goats and to evaluate the associated risk factors in Northwest Ethiopia.

## 2 Materials and method

### 2.1 Study area

**Figure 1:**
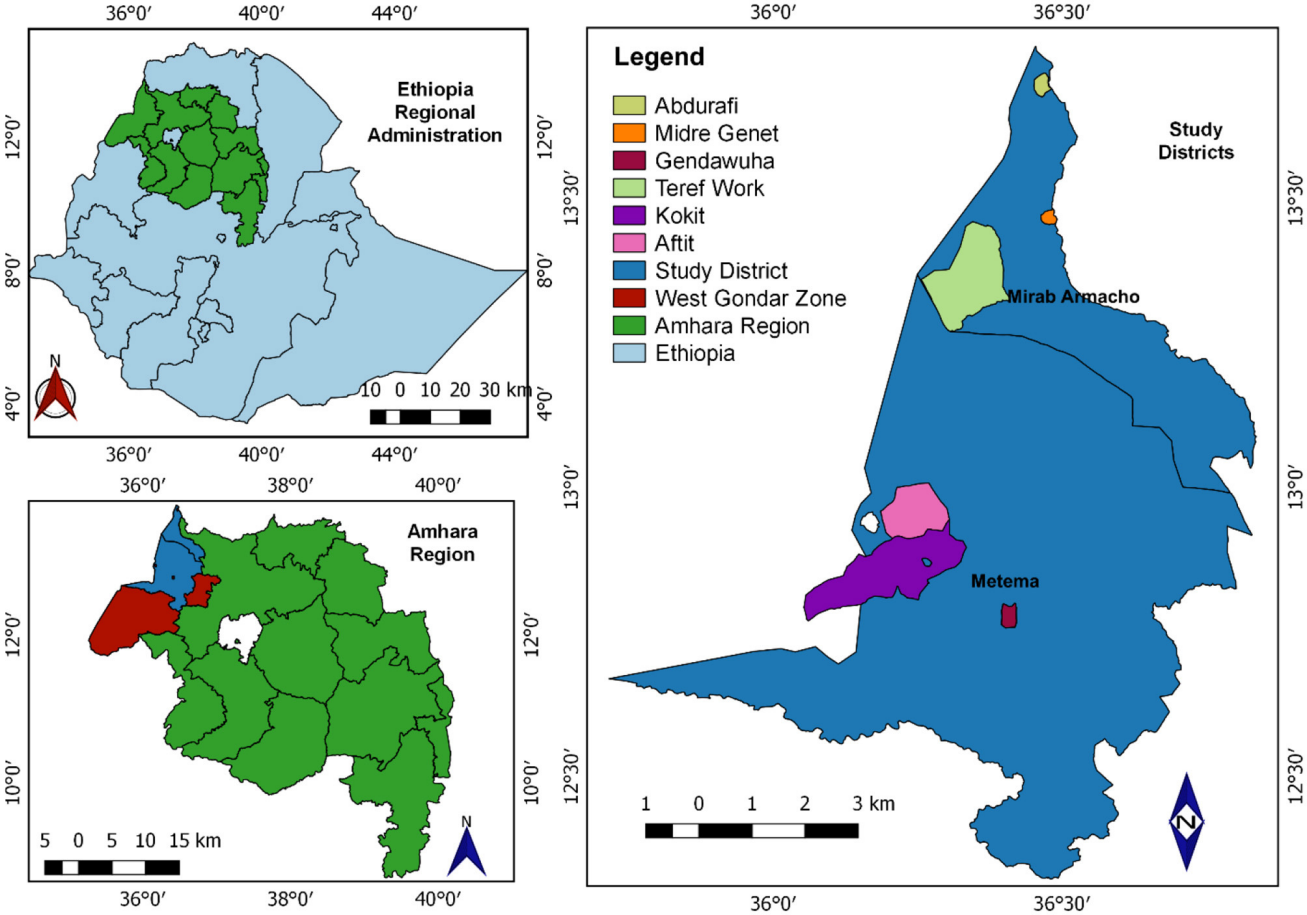
Map shows the Metenma and Mirab Armacho study districts (QGIS 3.22.6 software was used to draw).

In Ethiopia, there are nine regional states: Tigray, Afar, Amhara, Oromia, Southern Nations, Nationalities, and Peoples' Region (SNNPRS), Benishangul-Gumuz, Gambella, Harari, and Somali. The study was carried out in Northwest Ethiopia within the Amhara region, specifically in the West Gondar Zone. In the West Gondar Zone, the livestock populations consist of 4,677,125 cattle, 1,566,904 sheep, and 1,211,738 goats. Two districts, namely Metema and West Armacho were selected from West Gondar zone. Metema district receives annual rainfall ranging from 700 to 900 mm, and has mean annual minimum and maximum temperatures of 32 and 46°C, respectively. The altitude ranges from 500 to 700 m above sea level. Similarly, West Armacho district is positioned between latitude 13°00′00″ to 13°40′00″ North and longitude 36°20′00″ to 37°00′00″ East. The annual rainfall here varies from 600 to 1,100 mm, with mean annual minimum and maximum temperatures recorded at 30 and 45°C. The altitude in West Armacho district ranges from 500 to 700 m above sea level (Figure 1) (37).

### 2.2 Study design and period

A cross-sectional study was conducted from February 2023 to May 2023 in small ruminants.

#### 2.2.1 Study populations

The study animals were apparently healthy sheep and goats of all age, sex, and species found at different agro-ecological zones in the study area. The estimated animal population in Metema district was 537,981 cattle, 120,541 sheep and 288,933 goats. Similarly, the estimated animal population in West Armacho district was 1,522,758 cattle, 1,278,321 sheep and 139,953 goats. In both districts most part is covered by forest, bush and savannah grass, extensive grazing predominates and animals are not housed.

#### 2.2.2 Sample size determinations

The sample size was calculated using the method described by Tschopp et al. (38) and Dohoo et al. (39).


$$\begin{array}{l} n=gc=\frac{P\left(100-P\right)D}{SE^{2}} \end{array}$$

Where “n”: the sample size, “p”: the prevalence as a percentage, “D”: the design effect, “SE”: the standard error, “g”: the average number of individuals sampled per cluster, and “c”: the number of clusters.


$$\begin{array}{l} D=1+\left(g-1\right)ICC \end{array}$$

The estimate of intra-cluster correlation coefficient (ICC) for most infectious diseases does not exceed 0.2 (40). So, considering 0.2 ICC for the cluster (village) and the possibility of collecting about 18 serum samples per village (g), D equals 4.4. Sampling 18 animals per village with an expected prevalence of 50% and a standard error of 5% gave about 24 clusters, and thus a total sample size of 444.

#### 2.2.3 Sampling method

A multistage cluster sampling technique was employed to conduct the seroprevalence and risk factor study. Zones and districts were purposively selected based on the abundance of small ruminant populations and their proximity to Sudan. Specifically, two districts, West Armacho and Metema, were chosen from the West Gondar zone. Within each district, three kebeles (Kebele is the lowest level of local government and serves as a neighborhood-level administrative unit in Ethiopia) were selected using a simple random sampling technique: Gendawuha, Kokit, and Aftit kebeles from the Metema district, and Midre Genet, Abdurafi, and Teref Work kebeles from the West Armacho district. Villages within these kebeles were considered clusters, and four villages from each kebele were randomly selected. Individual animals from these villages were then randomly chosen to form the study units. The total sample collected was proportionally distributed among the selected clusters, resulting in a sample size of 444 animals, comprising 308 sheep and 136 goats. This systematic approach ensured a representative and balanced sample distribution across the study area, facilitating accurate assessments of BTV seroprevalence and associated risk factors.

### 2.3 Method of data collection

#### 2.3.1 Blood sample collection

About 5 ml of blood was collected from jugular veins using plain vacutainer tube and vacutainer needle after the site was cleaned, hair removed and disinfected with 70% alcohol. The collected blood samples then stand at 45° positions until the sera were collected and transported via ice box (+4°C) to the laboratory. The sera were stored in a refrigerator at −20°C until tested, at Veterinary Microbiology Laboratory at College of Veterinary Medicine and Animal Sciences, University of Gondar. During blood sample collection, supporting data were also collected using a relevant format.

#### 2.3.2 Potential risk factor

Structured data collection sheet was employed to collect information on geographical location, age, sex, breed and species of animal's sampled and flock size. Species (sheep and goats), age group was classified as [young (<1 year), adult (≤1 and ≤3 years) and older (>3 years)] as described by Yasine et al. (41) and Jemberu et al. (42), were recorded during sampling. The flock size was categorized into three groups depending on the number of small ruminants in the flock: small size ≤100), medium size (>100 and ≤200), and large size (<200) (43, 44). Information related to exposure to the putative risk factors of BTV such were included in the data collection sheet (Supplementary material 1).

### 2.4 Serological test

The competitive Enzyme-linked immunosorbent assay (cELISA) was used to discriminate BTV from another closely associated Orbivirus such as Epizootic Hemorrhagic Disease virus (EHDV). It was performed using BT antibody test kit (IDvet, 310, rue Louis Pasteur-Grabels France) following the procedures recommended by the manufacturer to the test is highly sensitive (100%) and specific (99%) serological tests (13, 45). The competition percentage (S/N%) and the cutoff were calculated and applied as recommended by the manufacturer, i.e., S/N% ≤ 70% = positive, 70% < S/N % < 80% = doubtful, S/N% ≥ 80% = negative. VP7 of BTV was targeted for antibody detection (anti-VP7) as described by Rojas et al. (46). The test was performed at National Veterinary Institute (NVI) as per the manufacturer's protocol

### 2.5 Data management and analysis

The collected data were entered into a Microsoft Excel spreadsheet. The data were then transferred and analyzed using Stata statistical software version 17. Descriptive as well as analytic statistics were used to summarize and analyze the data. Seroprevalence of BTV was computed by dividing the total number of seropositive sheep and goats by the total number of animals of each species sampled. The animal level true prevalence was calculated by adjusting the corresponding apparent seroprevalence (AP, as percentage) for 100% sensitivity (SE) and 99% specificity (SP) of c-ELISA for BTV (13) using the following formula as indicated by Dohoo et al. (39).


$$\begin{array}{l} \text{True prevalence}=\;\frac{\text{AP}+\text{SP}-100}{\text{SE}+\text{SP}-100} \end{array}$$

Associations between seropositivity (status of BTV infection in sheep and goats) and potential risk factors were initially examined using univariable analysis and chi-square analysis. Subsequently, a multivariable analysis was conducted utilizing a mixed-effects logistic regression model, with Kebele treated as a random effect variable. This model is employed to analyze binary outcome variables, where the log odds of the outcomes are modeled as a linear combination of both fixed and random effects. Only variables with a *p*-value < 0.5 were integrated into the multivariable model, to include the most important predictors that meaningfully contribute to the model. Moreover, it helps control confounding and enhances the practicality of decision-making. A multivariable model for the outcome variable was constructed using manual stepwise forward mixed effect logistic regression model. During the analysis confounding was checked and it was considered present if any of the remaining coefficients changed at least 25% after removing a non-significant (*p* > 0.05) variable from the model (47). Interactions were tested for all combinations of the significant main effects. Factors with a *p*-value < 0.05 in the final model were taken as a risk factor to BTV sero-prevalence.

## 3 Result

### 3.1 Seroprevalence of BTV

**Figure 2:**
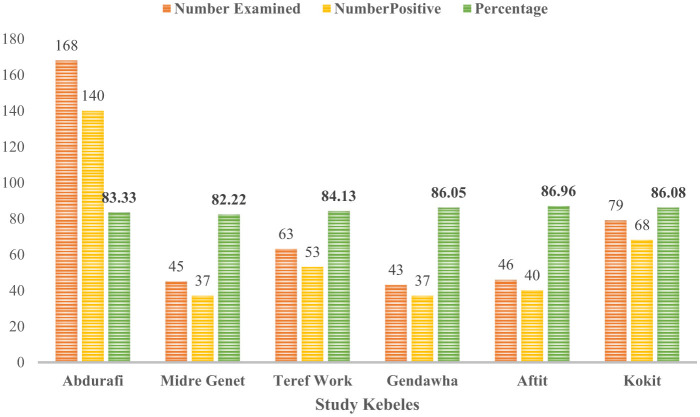
Seroprevalence of BTV in sheep and goats of different kebeles in West Gondar Zone Northwest Ethiopia.

The overall apparent sero-prevalence was 84.5% (*n* = 444; 95% CI: 80.8–87.5). Our study showed that higher apparent seroprevalence of BTV in sheep [83.4% (*n* = 308; 95% CI: 78.9–87.2)] and goats [86.8% (*n* = 136; 95% CI: 79.9–91.5)] (Table 1). In relation to Kebele, the seroprevalence of BTV antibodies was most prevalent in Aftit Kebele [86.96% (*n* = 46; 95% CI: 73.8–94.0)] and least in Midre Genet [82.2% (*n* = 45; 95% CI: 68.62–90.9)] (Figure 2).

**Table 1:** Seroprevalence of bluetongue virus in sheep and goats in the study districts.

**Variable**	**Category**	**No of animals tested**	**No of positive**	**Seroprevalence (%)**	**95% CI**
District	Metema	168	145	86.3	80.2–90.7
	W/Armacho	276	230	83.3	78.4–87.3
Species	Sheep	308	257	83.4	78.9–87.2
	Goat	136	118	86.8	79.9–91.5
Age	Young	96	61	63.5	53.5–72.6
	Adult	173	144	83.2	76.9–88.1
	Older	175	170	97.1	93.3–98.8
Sex	Male	130	99	76.2	68.1–82.7
	Female	314	276	87.9	83.8–91.2
Flock size	Small	84	68	80.9	71.1–88.0
	Medium	203	176	86.7	81.3–90.7
	Large	157	131	83.4	76.8–88.5
Housing	No	363	314	86.5	82.6–89.7
	Yes	81	61	75.3	64.8–83.5
MWOA	No	209	181	86.6	81.3–90.6
	Yes	235	194	82.6	77.1–86.9
Total		444	375	84.5	80.8–87.5

MWOA, mixed with other animals.

### 3.2 Risk factors for bluetongue serostatus

The study commenced with the execution of univariable analysis to examine the correlation between potential risk factors and BTV infection. The findings of the chi-square analysis have been delineated in Table 2. Subsequently, the variable was incorporated into a conclusive multivariable model and scrutinized using mixed effect logistic regression. During this evaluation, species and age emerged as statistically significant (*p* < 0.05). The final model revealed that older small ruminants were 25.95 times (95% CI: 9.45–71.28) and adult small ruminants 3.49 times (95% CI: 1.90–6.41) more likely to be seropositive with BTV compared to young small ruminants (Table 3). Moreover, there were no confounding and interactions in the final model. Only variables with a *p*-value < 0.5 were integrated into the final model.

**Table 2:** Chi-square analysis assessed association of potential risk factors for BTV seropositivity.

**Variable**	**Category**	**No of positives (%)**	**χ2**	***P*-value**
Species	Sheep	257 (83.4)	0.79	0.373
	Goats	118 (86.8)		
Age	Young	61 (63.5)	53.65	0.000
	Adult	144 (83.2)		
	Older	170 (97.1)		
Sex	Male	99 (76.2)	9.70	0.002
	Female	276 (87.9)		
Flock size	Small	68 (80.9)	1.69	0.430
	Medium	176 (86.7)		
	Large	131 (83.4)		
Housing	No	314 (86.5)	6.32	0.012
	Yes	61 (75.3)		
Altitude	>750	251 (82.8)	1.91	0.167
	<750	124 (87.9)		
MWOA	No	181 (86.6)	1.40	0.240
	Yes	194 (82.6)		

**Table 3:** Multivariable analysis of potential risk factors for BTV seropositivity in sheep and goats using mixed effect logistic regression models with random effect variable of kebele.

**Variable**	**Category**	**AOR**	**95% CI**	***P*-value**
Species	Sheep	Ref.		
	Goats	2.43	1.27–4.62	0.007
Age	Young	Ref.		
	Adult	3.49	1.90 −6.41	0.001
	Older	25.95	9.45– 71.28	0.001

AOR, adjusted odd ratio; MWOA, mixed with other animals; Ref, reference; CI, confidence interval.

## 4 Discussion

The presence of bluetongue virus antibodies was detected in sheep and goats. The identification of antibodies against the bluetongue virus in Northwest Ethiopia signifies the endemic nature of bluetongue virus sero-prevalence among sheep and goats in the Northwest Ethiopia (18).

The seroprevalence of BTV (84.5%) identified in the current investigation aligns closely with the prevalence reported (84.6%) by Elhassan et al. (48) in Sudan. While the prevalence observed in this study is relatively high, it is lower compared to findings by Shoorijeh et al. (49) in Iran (93.5%), Najarnezhad and Rajae (50) in Iran (89.2%), Elmahi et al. (18) in Sudan (91.2%), and Gür (51) in Southern Turkey (88%). Conversely, the current findings demonstrate a higher seroprevalence of BTV in small ruminants compared to previous reports by various authors across different countries: 41.17% in small ruminants in Southern Ethiopia (36), 53.3% in sheep in India (10), 78.4% in small ruminants in Grenada (52), 56.6% in sheep in Pakistan (53), 55.3% in small ruminants in Bangladesh (54), 28.26% in sheep in Brazil (55), 54.10% in sheep in Saudi Arabia (56), and 67.7% in goats in Iran (57). The seroprevalence rates of 86.8% in goats and 83.4% in sheep documented in this study align with the findings of Mohammadi et al. (58), who reported seropositivity rates of 74.2 and 72.9% for goats and sheep, respectively, in Fars Province, Iran. However, the present findings are higher than those reported by Medrouh et al. (17) in Africa, who observed seroprevalence rates of 36.3% in sheep and 47.0% in goats, as well as by van den Brink et al. (59) in the Netherlands, who reported a rate of 7.0% in sheep. The observed disparities in seropositivity levels could be attributed to variations in sample size, study duration, geographical location, immune status, climatic conditions, husbandry practices, management strategies, and vector control interventions implemented around the different study areas. Additionally, it is well-documented that goats, even with minimal clinical signs, can harbor high levels of BTV, potentially serving as a source of infection for other susceptible animals (60, 61). BTV possesses multiple serotypes and immunity to one serotype offers little cross-protection to other serotypes (62).

The higher seropositivity rate among older animals (97.1%) aged over 3 years, compared to younger animals (63.5%) under 1 year of age, as observed in this study, aligns with the findings of Puri et al. (63), who reported a higher seropositivity rate among older animals (49.12%) over 6 months old, compared to younger animals (16.32%) under 6 months old in Nepal. Similarly, a systematic review and meta-analysis conducted by Medrouh et al. (17) in Africa, as well as a study by Ferrara et al. (64) in Italy, reported a higher seroprevalence rate of 46.2 and 46.0%, respectively, among adult animals. This difference can be attributed to several factors. Younger animals are typically sheltered indoors and receive attentive care from their owners, practices that help prevent insect and tick-borne infections. Notably, it was observed that younger animals became infected with BTV when they commenced grazing in the fields at 6 months of age (65). The variation in prevalence across age groups is likely due to the increased exposure of older animals to bluetongue virus infections over their longer lifespan, making them more susceptible to the disease.

The present investigation demonstrated a relatively higher seropositivity among females (87.9%) compared to males (76.2%). This finding aligns with previous studies, including research by Gizaw et al. (1) in Ethiopia, which reported a higher seroprevalence in females (65.54%) compared to males (49.85%). Similarly, Medrouh et al. (17) observed a greater seroprevalence in females (53.3%) than in males (28.1%) in Africa. Additionally, a study by Puri et al. (63), in Nepal found a higher seroprevalence in females (45.6%) compared to males (26.09%). These consistent findings suggest a potential sex-based difference in susceptibility or exposure, warranting further investigation. Moreover, the discrepancy might be attributed to a potential sampling bias, as more female ruminants were included in the current study, similar to findings from a previous study by Elhassan et al. (48). The study also revealed that BTV infection rates increase with larger flock sizes: small flocks (80.9%), medium flocks (86.7%), and large flocks (83.4%). This observation aligns with the findings of Haile et al. (12), who reported seroprevalence rates of 37.42% for small flocks, 32.35% for medium flocks, and 64.91% for large flocks, as well as with the findings of Sana et al. (66), who reported rates of 36.95% for small flocks, 40% for medium flocks, and 43.59% for large flocks. However, the current results contrast with the findings of Munmun et al. (65), who documented prevalence rates of 58.33% in small flocks, 32.79% in medium flocks, and 38.46% in large flocks. This discrepancy may be due to differences in environmental conditions, vector populations, agro-ecological factors, and management practices that influence interactions with the vector of the bluetongue virus in different regions.

In the current study, older and adult small ruminants were found to be 25.95 times (95% CI: 9.45–71.28) and 3.49 times (95% CI: 1.90–6.41) more likely to be infected with BTV compared to younger small ruminants. This finding is consistent with previous reports indicating that adult small ruminants had higher odds of infection: 2.97 times (95% CI: 1.88–4.69) in Ethiopia (13), 1.76 times (95% CI: 1.09–2.85) in Ethiopia (36), 4.30 times (95% CI: 1.94–9.57) in Sudan (67), 1.48 times (95% CI: 1.12–1.9) in Italy (64), 2.41 times (95% CI: 2.13–2.74) and 2.43 times (95% CI: 2.15–2.75) in Iran (68), and 1.73 times (95% CI: 1.73–1.42) and 4.68 times (95% CI: 3.79–5.78) in Iran (69). The discrepancies in the odds of BTV occurrence observed across different studies may be attributed to variations in management practices and environmental factors. The higher seroprevalence observed in older animals is likely attributable to their prolonged exposure to the BTV over time. Additionally, differences in immune response between age groups may play a role, with older animals potentially developing higher levels of antibodies due to repeated or prolonged exposure to the virus. This phenomenon may be attributed to protective measures such as indoor housing and careful management by owners, which help shield young animals from *Culicoides* midge bites and other infections. In addition to these, a study by Ayele et al. (31) on *Culicoides* vector identification and spatial distribution demonstrated that the study area (Northwest Ethiopia) had a high abundance of *Culicoides* and provided a more favorable environment for the vector compared to findings from similar studies in Southern Ethiopia by Fetene et al. (32). Previous studies have documented that young animals become infected with BTV when introduced to grazing fields at 6 months of age (36). However, conflicting results were reported by Sana et al. (66) in Tunisia and Munmun et al. (65) in Bangladesh, which do not align with the current findings.

Moreover, a species that was not statistically significant in the univariable model became significant in the final multivariable model. This suggests that the effect of the species was initially masked by other variables in the univariable analysis. However, after adjusting for these variables in the multivariable model, the contribution of the previously non-significant species became more apparent. In this study, goats were found to be 2.43 times (95% CI: 1.27–4.62) more susceptible to infection compared to sheep, which is consistent with findings from Islam et al. (54) in Bangladesh (AOR = 4.69, 95% CI: 2.49–8.82) and Manavian et al. (69) in Iran (AOR = 0.43, 95% CI: 0.37–0.50). However, the results reported by Bakhshesh et al. (68) in Iran (AOR = 1.03, 95% CI: 0.97–1.10) and Yilma and Mekonen (36) in Ethiopia (AOR = 1.17, 95% CI: 0.68–2.01) did not align with the findings of this study. This variability could be due to differences in environmental conditions, management practices, or other unidentified factors influencing the susceptibility of these species to BTV.

This study faced several limitations that should be considered when interpreting the findings. First, the inability to identify the specific serotypes circulating in the study area posed a significant challenge. This limitation was primarily due to resource constraints, including inadequate laboratory facilities and insufficient funding. Identifying specific serotypes is essential for understanding disease epidemiology, designing effective control measures, and assessing vaccine suitability. Second, the overrepresentation of goats in the sample, although they are a key livestock species in the region, may limited the generalizability of the findings to other species, such as cattle and sheep. This underscores the importance of future studies employing more representative sampling strategies to provide a comprehensive epidemiological perspective. Similarly, herd size data were collected as categorical variables, and intra-flock seroprevalence was not reported; therefore, we acknowledge these as limitations. This underscores the need for future studies with an intra-flock level seroprevalence report.

## 5 Conclusion and recommendation

The current investigation disclosed a notable seroprevalence of 84.5% and the endemicity of BTV among sheep and goats within the designated area under study. Age and species were identified in this study as potential risk determinants for Bluetongue Virus infection in sheep and goats. A higher seroprevalence of BT was observed in goats, particularly in adult and older small ruminants, in comparison to sheep and younger small ruminants, respectively. Consequently, upcoming investigations concerning this disease should prioritize the identification of prevalent serotypes and circulating vector species.

## Data Availability

The raw data supporting the conclusions of this article will be made available by the authors, without undue reservation.
