# Supplementary material for: Seroprevalence and risk factors of bluetongue virus infection in sheep and goats in West Gondar zone, Northwest Ethiopia
Source: Front Vet Sci. 2025 Mar 5;12:1565624. doi: 10.3389/fvets.2025.1565624 (PMC11920756; doi:10.3389/fvets.2025.1565624)
Supplement: Supplementary file 1 [file Data_Sheet_1.pdf]

Supplementary File 1: Data collection format for serological analysis of BTV

District----------------Kebele-------------Village-------------Altitude -------------Season----------Lat----------Long-------

Small ruminant number/village-------- Average envi. temperature--------Rainfall ---------Vegetation type: ------------

Ani. ID	Species	Sex	Breed	Age	Management system	Origin	Mixed with other livestock	Herd size	Housing

Vegetation type: bush land, forest, grass land, cultivated land; **Species=** sheep/goat; **Sex** =male/female; **Breeds**=cross/local; **Management system**= restricted/ free grazing; **Origin**= born in the herd/brought in; **Mixed with other livestock**= yes/no, **Herd size**= $\leq$ 100, >100 and $\leq$200 and > 200; **Housing**= yes/no
